# A Flexible Capacitive Humidity Sensor Enabled by LIG-Anchored Synergistic GO-PEDOT:PSS-MXene Composite

**DOI:** 10.3390/ma19122537

**Published:** 2026-06-11

**Authors:** Jitong Ren, Ronghui Dan, Yanyan Guo, Jiang Zhao

**Affiliations:** College of Integrated Circuit Science and Engineering, Nanjing University of Posts and Telecommunications, 9 Wenyuan Road, Nanjing 210023, China

**Keywords:** capacitive, humidity sensor, graphene oxide, PEDOT:PSS, MXene, laser-induced graphene

## Abstract

Indispensable roles in personalized health monitoring and human–machine interaction are played by flexible humidity sensors. However, high costs and complex vacuum processes are often involved in current fabrication methods, thereby restricting their broader applications. In this work, a high-performance flexible capacitive humidity sensor is presented, wherein a ternary composite of graphene oxide, PEDOT:PSS, and MXene (GO-PEDOT:PSS-MXene) is loaded onto a laser-induced graphene (LIG) interdigitated electrode. A pronounced synergistic effect among the three components is systematically exploited by this multidimensional architecture to significantly optimize the overall sensing performance. Within a relative humidity range extending from 11% to 97%, a remarkable measurement sensitivity of 18,643.02 μF/%RH is recorded. Furthermore, a characteristic negative capacitive response is consistently induced by moisture-driven microstructural swelling, by which the internal interlayer spacing is increased. The continuous monitoring of human respiratory rhythms and precise non-contact spatial sensing is successfully enabled by rapid response and recovery times of 31.7 s and 11.2 s, respectively. Uniquely, a vacuum-free, synergistic multidimensional architecture is successfully utilized to achieve an ultrahigh sensitivity. Practically, a highly scalable and low-cost paradigm is established by this research for the mass deployment of future wearable electronic systems across diverse monitoring scenarios.

## 1. Introduction

Flexible humidity sensors represent a core technology for the accurate monitoring of environmental humidity. Substantial promise is also shown by these devices across developing domains like personal health monitoring [1], environmental control [2], soft robotics [3], and human–machine interaction [4]. Conventional humidity sensors, however, are typically constructed on rigid substrates and possess large form factors. This construction severely impedes their seamless integration into flexible and wearable electronics. Among various sensing mechanisms, including resistive [5,6], capacitive [7,8], and piezoelectric [9,10] types, flexible capacitive humidity sensors emerge as a leading approach due to superior overall performance. Their high sensitivity enables the precise detection of minute changes in the dielectric constant. Furthermore, they demonstrate excellent long-term stability, low temperature dependence, rapid response/recovery times, and an expansive detection window [11]. Within the domain of advanced humidity monitoring, capacitive flexible sensors serve as the preferred technology due to their intrinsic benefits.

As the field of flexible electronics continues its rapid evolution, attributes of high mechanical compliance and amenability to scalable production are necessary for sensing devices. Conventional flexible metal microelectrodes, typically using gold, silver, copper, or platinum, face significant challenges related to cost and fabrication complexity. Their production commonly relies on cumbersome processes such as magnetron sputtering [12] or vacuum evaporation [13]. These methods require expensive raw materials, stringent cleanroom environments, and large-scale equipment, which substantially increase the manufacturing cost of high-performance flexible sensors and limit their commercialization. To overcome this bottleneck, polyimide (PI) film stands out as an ideal flexible substrate due to its exceptional thermal stability, chemical resistance, and biocompatibility. More importantly, the formation of a porous graphene network, identified as laser-induced graphene (LIG), from PI film is rapidly accomplished in situ under ambient conditions through the utilization of laser scribing technology [14,15,16]. This technique circumvents the limitations of traditional photolithography masks, allowing for flexible electrode patterning and high scalability [17]. The LIG approach thus provides a highly promising paradigm for the low-cost, large-scale manufacturing of electrodes for flexible humidity sensors.

The microstructure and physicochemical properties of the sensing material fundamentally govern sensor performance. Polymers [18,19], metal oxides [20,21], two-dimensional (2D) materials [22,23], and composites [8,24] have all been extensively explored for humidity sensing applications. Among these, PEDOT:PSS, a highly conductive polymer, attracts considerable attention due to its excellent hygroscopic properties [25]. Similarly, 2D graphene oxide (GO) features an extensive specific surface area and a high density of oxygen-based moieties, exhibiting high affinity for water molecules and facilitating their rapid permeation [26]. Nevertheless, single-material or simple binary composite systems often encounter significant limitations. The strong hygroscopicity of PEDOT:PSS tends to induce film swelling, which compromises long-term device stability. The planar morphology of GO nanosheets makes them vulnerable to interlayer aggregation, which consequently leads to a substantial decrease in effective surface area and creates barriers to the internal diffusion of water molecules. To address these deficiencies and further advance sensing performance, the incorporation of 2D transition metal carbides/nitrides (MXenes) into a ternary composite system emerges as a breakthrough strategy [27,28]. MXenes are characterized by their remarkably high electrical conductivity, on par with metallic materials, coupled with surface functionalization comprising hydrophilic terminations (–OH, –O, and –F) that provide favorable sites for water molecule sequestration. The integration of GO, PEDOT:PSS, and MXene into a unified architecture yields a remarkable multidimensional synergistic effect. The intercalation of 2D MXene and GO nanosheets generates pronounced steric hindrance, effectively preventing restacking and maintaining expanded interlayer spacing that provides unobstructed diffusion pathways for water molecules. Concurrently, PEDOT:PSS interpenetrates the 2D nanosheet scaffold and, together with the highly conductive MXene, forms a robust and efficient three-dimensional electron transport network. Additionally, the rich hydrophilic groups of the three components couple through an extensive hydrogen bond network, substantially enriching the active adsorption sites at the sensing interface. This configuration produces a significant synergistic enhancement, establishing a superior mechanism for both water molecule capture and charge conversion.

Despite the promise of these advanced nanocomposites, existing flexible humidity sensors predominantly rely on expensive metal microelectrodes fabricated via complex vacuum processes, and they typically operate on conventional positive dielectric polarization mechanisms. Therefore, a significant research gap remains in developing fully scalable, low-cost architectures that exploit novel mechanosensitive responses for enhanced performance. To address this gap, the present work reports the successful construction of a novel flexible capacitive GO-PEDOT:PSS-MXene@LIG humidity sensor. A high-performance ternary composite sensing gel is obtained through the uniform dispersion of GO, PEDOT:PSS, and MXene solutions. Laser direct writing is employed to fabricate flexible LIG interdigitated electrodes with high efficiency. Subsequently, a facile drop-casting and thermal treatment process enables intimate integration of the sensing layer with the LIG electrodes. Experimental results demonstrate that the as-prepared humidity sensor delivers exceptional sensing performance across a wide relative humidity (RH) range. Herein, we systematically evaluate the device’s multi-scenario capabilities, validating its performance boundaries as an integrated platform for physiological metrics and non-contact spatial tracking.

## 2. Experimental

### 2.1. Materials

GO solution (2 mg mL^−1^) was procured from Suzhou Carbonfeng Technology Co., Ltd., Suzhou, China. PEDOT:PSS solution containing 1.1–1.3 wt% solid content was acquired from Agfa-Gevaert N. V., Mortsel, Belgium. MXene solution at 5 mg mL^−1^ concentration was provided by Nanjing XFNANO Tech Co., Ltd., Nanjing, China. The full set of inorganic salts adopted in this study was procured from two qualified commercial chemical manufacturers in China. Specifically, LiCl, CuCl_2_, and NaCl were sourced from Sinopharm Chemical Reagent Co., Ltd., Shanghai, China, while CH_3_COOK, MgCl_2_, K_2_CO_3_, NaBr, KCl, and K_2_SO_4_ were supplied by Shanghai Aladdin Biochemical Technology Co., Ltd., Shanghai, China. Meanwhile, a Kapton-type PI film with a thickness of 100 μm was obtained from Guangzhou Beilong Electronic Materials Co., Ltd., Guangzhou, China. For the entire experimental workflow, highly purified water was employed for all relevant operations, and all reagents used were of analytical grade, with no additional purification performed prior to use.

### 2.2. Sensor Fabrication Process

**Figure 1:**
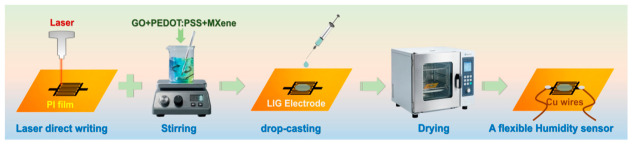
Schematic representation of the flexible GO-PEDOT:PSS-MXene@LIG humidity sensor.

The fabrication process of the flexible GO-PEDOT:PSS-MXene@LIG humidity sensor is depicted in Figure 1. LIG interdigitated electrodes were generated in situ on a 100 µm thick PI film, which served as the substrate. The process involved laser direct scribing (power: 16 W, speed: 90 mm s^−1^) at room temperature and atmospheric pressure, conforming to a predefined electrode pattern, the dimensional schematic and photograph of which are detailed in Figure S1. In parallel, the humidity-sensitive functional material was prepared through the following procedure: A base solution was first established by diluting the stock PEDOT:PSS solution to one-tenth of its original concentration. A GO-PEDOT:PSS composite dispersion was then formed by the dropwise addition of 3 mL of a 2 mg mL^−1^ GO solution to 30 mL of this base solution, followed by ultrasonication in a water bath for 10 min to ensure thorough mixing. Subsequently, 0.2 mL, 0.5 mL, 1 mL, and 2 mL of MXene dispersion (5 mg mL^−1^) were separately added to the above mixtures, followed by a second water-bath ultrasonication treatment for 10 min to obtain uniformly dispersed GO-PEDOT:PSS-MXene ternary composite humidity-sensitive materials. To finalize the deposition of the humidity-sensitive functional gel, the effective sensing area of the LIG interdigitated electrode was coated with a precise 0.2 mL volume of the composite dispersion via drop-casting, followed by a one-hour drying process at 60 °C. Finally, copper wires were connected to the patterned LIG electrode via conductive silver paste through ohmic contact. Following the final curing step, the successful fabrication of a flexible GO-PEDOT:PSS-MXene@LIG humidity sensor was achieved.

### 2.3. Characterization and Measurement

The surface and internal microstructures of the electrode material and the humidity-sensitive material were characterized by means of Scanning Electron Microscopy (SEM, MIRA LMS, TESCAN, Brno, Czech Republic) and Transmission Electron Microscopy (TEM, JEM-2100Plus, JEOL, Akishima, Japan). An understanding of the materials’ chemical states and electronic structures was achieved with data from X-ray Photoelectron Spectroscopy (XPS, Thermo Scientific K-Alpha, Waltham, MA, USA) and a confocal Raman spectrometer (HORIBA LabRAM HR Evolution, Palaiseau, France) using a 532 nm laser source.

To establish stable, controlled RH environments at 25 °C, various saturated salt solutions (LiCl, CH_3_COOK, MgCl_2_, K_2_CO_3_, NaBr, CuCl_2_, NaCl, KCl, and K_2_SO_4_) were prepared and maintained inside sealed glass vessels. Utilizing their distinct thermodynamic vapor-liquid equilibria, these solutions provided a comprehensive operational range of 11%, 23%, 33%, 43%, 58%, 68%, 75%, 85%, and 97% RH, respectively, for experimental evaluation (Figure S2).

The performance of the humidity sensor was quantified by its sensitivity (*S*). This parameter was calculated using the following equation: *S* = (*C_max_* − *C_min_*)/(*RH_max_* − *RH_min_*) × 100%. Here, *C_max_* and *C_min_* corresponded to the maximum and minimum measured capacitance values, respectively, while *RH_max_* and *RH_min_* represented the upper and lower bounds of the RH range. The electrical properties of the sensor were characterized using a Tonghui TH2832 LCR meter (Changzhou, China) with an AC voltage of 1 V (100 Hz). Electrochemical impedance spectroscopy (EIS) measurements were performed using a DH7003B dual-channel electrochemical workstation (Jiangsu Donghua Analytical Instrument Co., Ltd., Jingjiang, China).

During the preparation of the graphical abstract, the authors used Gemini 3.1 Pro to assist with image refinement. The authors reviewed and edited the output and take full responsibility for the final content.

## 3. Results and Discussion

### 3.1. Microstructural and Morphological Characterization

**Figure 2:**
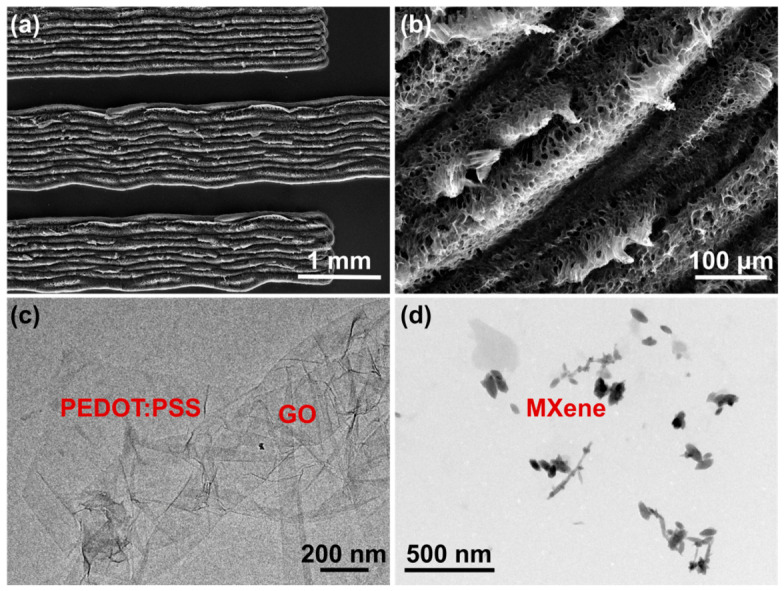
Morphological characterization of the electrode and the humidity-sensitive material. (**a**,**b**) SEM images of LIG interdigitated electrodes at high and low magnifications. (**c**,**d**) TEM images of GO-PEDOT:PSS-MXene, (**c**) GO-PEDOT:PSS, and (**d**) MXene.

SEM characterizes the surface morphology and microstructure of the LIG electrode. Figure 2a clearly shows that the interdigitated lines of the LIG electrode exhibit distinct features, with each digit displaying parallel stripes that align closely with the laser etching direction. Further observation (Figure 2b) reveals the porous characteristics of the LIG electrode and the wrinkled three-dimensional (3D) porous graphene nanosheets, which likely result from gas release during the laser irradiation process.

TEM is employed to systematically characterize the microscopic morphology and multiphase interfacial structure of the GO-PEDOT:PSS-MXene ternary composite humidity-sensing material. As shown in Figure 2c, GO exhibits a typical 2D flexible wrinkled lamellar structure, with the lamellae interconnecting to form a continuous network skeleton. PEDOT:PSS adheres closely to the GO lamellar surfaces in the form of a uniform and compact amorphous thin film. Figure 2d clearly reveals that numerous spindle-shaped and short-rod-shaped MXene nanoparticles with uniform size distribution are dispersed throughout the composite matrix, with only minimal agglomeration resulting from van der Waals forces. Such dispersed MXene nanoparticles synergistically cooperate with GO and PEDOT:PSS to construct a multidimensional conductive network. The abundant polar functional groups on the MXene surface serve to augment the hydrophilicity of the composite material, thereby establishing a structural foundation for superior humidity-sensing performance.

**Figure 3:**
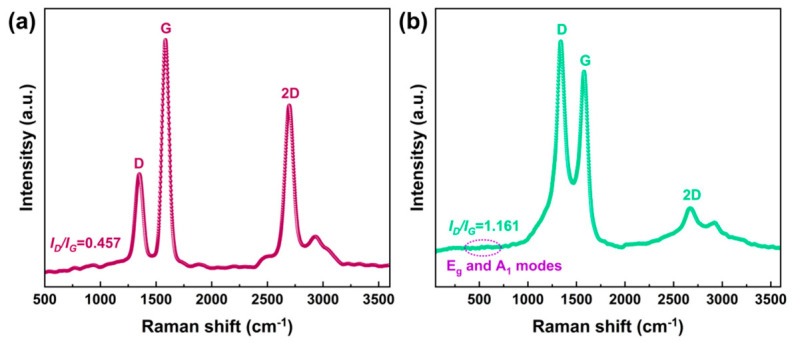
Raman spectra. (**a**) LIG. (**b**) GO-PEDOT:PSS-MXene.

Raman spectroscopy, as a non-destructive analytical technique, effectively reveals the microstructural characteristics of carbon-based materials. As shown in Figure 3a, the LIG electrode exhibits three typical characteristic peaks: the D peak at 1349.6 cm^−1^ originates from structural defects and disorder; the G peak at 1584.6 cm^−1^ corresponds to the stretching vibration of sp^2^-hybridized carbon and serves as a key indicator for assessing the degree of graphitization [29]; the prominent 2D peak at 2696.7 cm^−1^ is associated with double-resonance Raman scattering, indicating that laser induction generates multilayer graphene structures [30]. Since the relative intensity between the defect peak and the graphitization peak (*I_D_*/*I_G_*) exhibits a relationship inversely proportional to the level of graphitization, the extremely low *I_D_*/*I_G_* value (0.457) of LIG confirms the formation of a highly ordered graphitized carbon network within its structure. For the GO-PEDOT:PSS-MXene humidity-sensitive material, its Raman spectrum (Figure 3b) also exhibits the D band (1341.1 cm^−1^), G band (1580.4 cm^−1^), and 2D band (2667.4 cm^−1^). Distinctively, this material also presents A_1g_ and E_g_ mode characteristic peaks in the low-frequency region below 800 cm^−1^, induced by Ti and C atoms [31]. Furthermore, the *I_D_*/*I_G_* value of this humidity-sensitive material increases to 1.161, indicating the presence of relatively pronounced defects in the graphene layers within its structure.

**Figure 4:**
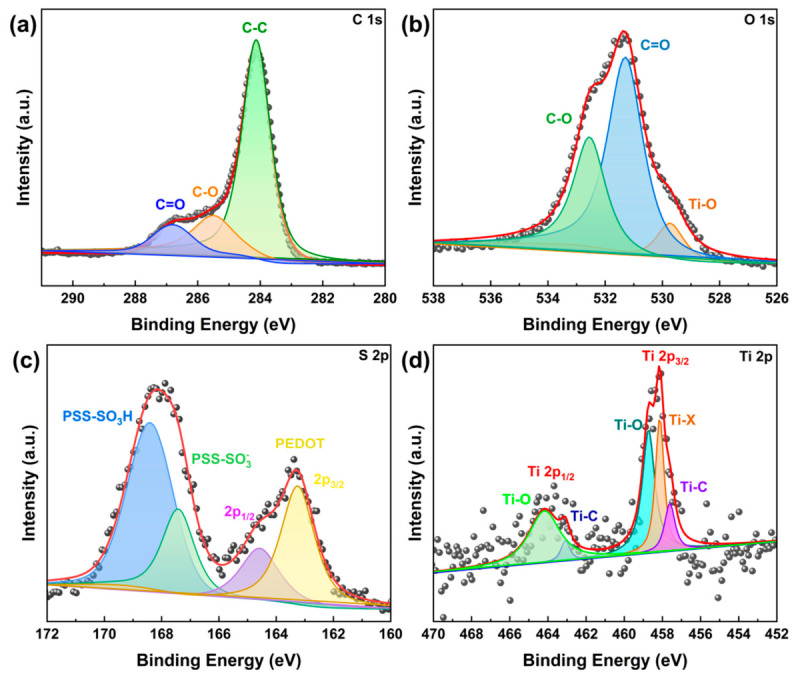
High-resolution XPS spectra of GO-PEDOT:PSS-MXene. (**a**) C 1s, (**b**) O 1s, (**c**) S 2p, and (**d**) Ti 2p.

To investigate the surface chemical properties and chemical bonding configurations of the GO-PEDOT:PSS-MXene, XPS characterization is performed. As shown in Figure 4a, the high-resolution C 1s spectrum is deconvoluted into three distinct components corresponding to C−C, C−O, and C=O bonds with binding energies at 284.2, 285.6, and 286.7 eV, respectively [32]. The O 1s spectrum presented in Figure 4b similarly consists of three deconvoluted peaks at 529.7, 531.3, and 532.6 eV, which are assigned to Ti−O, C=O, and C−O bonds, respectively [33]. For sulfur, the high-resolution S 2p spectrum (Figure 4c) reveals the structural characteristics of PSS: the absorption peak at 167.4 eV originates from the sulfonate groups on the PSS segments, while the peak appearing near 168.4 eV confirms the presence of -SO_3_H groups [34]. Additionally, the S 2p_3/2_ and S 2p_1/2_ orbital spin-split peaks of sulfur atoms in the PEDOT backbone are precisely located at 163.2 eV and 164.6 eV [35]. In the Ti 2p spectrum shown in Figure 4d, two major characteristic regions associated with Ti 2p_3/2_ and Ti 2p_1/2_ orbitals are observed. Further peak-fitting analysis indicates that the Ti 2p_3/2_ band contains three sub-peaks located at 457.6 eV (Ti−C), 458.2 eV (Ti−X), and 458.7 eV (Ti−O). Correspondingly, the Ti 2p_1/2_ band is resolved into two independent signal peaks at 463.1 eV (Ti−C) and 464.2 eV (Ti−O) [36].

### 3.2. Device Performance

The blending ratio of the GO-PEDOT:PSS binary matrix is adopted from our previous optimization framework [27]. Based on this foundation, various MXene contents are introduced to determine the optimal ternary composite formulation. As shown in Figure S3, comparison of device performance with different MXene additions reveals that the incorporation of 0.5 mL MXene exhibits the highest humidity-sensing sensitivity. Consequently, this specific composition is established as the optimal formulation and employed in all subsequent experiments.

**Figure 5:**
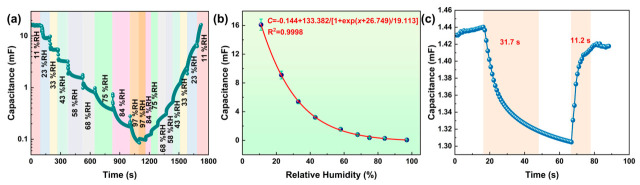
Humidity-sensing capabilities exhibited by the GO-PEDOT:PSS-MXene@LIG humidity sensor. (**a**) Hysteresis loop curves of dynamic response and recovery. (**b**) Capacitive response of the sensor as a function of RH (n = 3 independent devices; error bars represent standard deviation). (**c**) Response and recovery times.

Figure 5a displays the dynamic hysteresis loop of the GO-PEDOT:PSS-MXene@LIG sensor evaluated via sequential humidification and dehumidification cycles across the 11–97% RH range. The capacitive response exhibits a consistent, monotonic stepwise drop during moisture exposure that mirrors its recovery path during the drying phase. This high consistency between the forward and reverse testing phases confirms the sensor’s exceptional reversibility and dependable wide-range tracking capabilities. Furthermore, based on the hysteresis curve (Figure S4), the hysteresis value of the sensor is calculated using the formula *H* = ±Δ*H_max_*/(2*F_RH_*), where Δ*H_max_* represents the maximum capacitance variation between response and recovery phases, and *F_RH_* denotes the capacitance fluctuation within the 11% to 97% RH range [37]. The results show that the sensor exhibits a hysteresis value of approximately 9.41%, demonstrating excellent low-hysteresis characteristics that fully validate its suitability for practical applications.

Figure 5b details the capacitive response of the GO-PEDOT:PSS-MXene@LIG humidity sensor as a function of RH and characterizes the sensor’s sensitivity to moisture variations. Over the RH range of 11–97%, the sensor capacitance decreases monotonically with increasing humidity, indicating high sensitivity to humidity changes. Calculation based on the definition of *S* yields a value of 18,643.02 μF/%RH within this operational range. Further analysis reveals that the sensor capacitance (*C*) as a function of relative humidity (*x*) can be accurately fitted by *C* = 0.144 + 133.382/[1 + exp ((*x* + 26.749)/19.113)], yielding an excellent fit with R^2^ = 0.9998, which provides a mathematically robust foundation for quantitative calibration of the humidity sensor.

The time required for a sensor to reach 90% of its peak response during adsorption is designated as the response time, while the period needed to return to the 90% threshold during desorption is known as the recovery time. As depicted in Figure 5c for the GO-PEDOT:PSS-MXene@LIG humidity sensor, these characteristic times were calculated to be 31.7 s and 11.2 s, respectively.

Long-term stability, mechanical flexibility, and reproducibility are paramount metrics for flexible humidity sensors. The GO-PEDOT:PSS-MXene@LIG humidity sensor undergoes continuous stability testing over seven days under both low and high humidity conditions. The sensor exhibits excellent stability throughout the entire testing period (Figure S5a). To evaluate mechanical flexibility, humidity response curves are measured at different bending angles (30°, 60°, and 90°) at 58% RH (Figure S5b). The sensing performance remains highly consistent across all bending angles, demonstrating the robust mechanical flexibility of the device. To verify reproducibility, three independent batches of devices are fabricated using identical protocols and tested at 58% RH (Figure S5c). For highly sensitive capacitive sensors, manual fabrication inevitably introduces slight variations, resulting in minor differences in the capacitance response curves among the three batches. Nevertheless, the overall humidity sensing performance displays substantial consistency across all batches.

Collectively, the comparative data in Table S1 show that the as-prepared GO-PEDOT:PSS-MXene@LIG humidity sensor offers significant advantages in sensitivity and operational humidity range over other reported capacitive humidity sensors.

### 3.3. Sensing Mechanism

Analysis of the dynamic evolution of Nyquist plots from 11% RH to 97% RH (Figure S6) profoundly reveals the intrinsic mechanism underlying this negative capacitance response from an electrochemical impedance perspective. In AC impedance theory, the equivalent parallel capacitance *Cp* is mathematically derived from the real part *Z*′ (resistive characteristic) and imaginary part *Z*″ (capacitive characteristic) of complex impedance through the following equation:

(1) $$C_{p}=\frac{-Z^{\prime \prime }}{\omega \left({Z^{\prime }}^{2}+{Z^{\prime \prime }}^{2}\right)}$$

At 11% RH, the impedance spectrum contracts extremely close to the left baseline (*Z*′ approximately 90.5 Ω), forming almost no distinct impedance semicircle, with the imaginary part -*Z*″ remaining very close to zero. Under this condition, the inherent series resistance *R_s_* ≈ 90 Ω dominates the system. In the absence of water molecule interference, MXene, PEDOT:PSS, and LIG constitute a perfect, seamlessly interconnected 3D highly conductive percolation network [38,39], rendering the bulk resistance (*R_film_*) within the sensing layer nearly zero. In this state, the entire sensing layer functions as a highly conductive electrode with an ultrahigh specific surface area, enabling unimpeded rapid charge accumulation at microscopic interfaces and establishing a substantial electric double-layer capacitance or pseudocapacitance. The extremely small impedance modulus in the denominator consequently yields an exceptionally high initial capacitance at the macroscopic level. As RH increases progressively, the diameter of the semicircular impedance arc in the Nyquist plot exhibits explosive expansion. The real-axis intercept *Z*′ extends rapidly rightward (surging from approximately 90 Ω to approximately 138 Ω), while the peak value of the imaginary axis -*Z*″ simultaneously elevates substantially. The diameter of the impedance arc essentially represents the charge transfer resistance or *R_film_* of the sensing layer. The dramatic enlargement of the impedance arc indicates a significant increase in internal device resistance. Substituting this impedance variation into the capacitance calculation formula directly demonstrates how high-resistance barriers eliminate capacitance: at elevated humidity, the real impedance *Z*′ increases markedly. In Equation (1), *Z*′ appears in squared form (*Z’*^2^) within the denominator, and its dramatic growth exerts a strong mathematical suppression effect on the overall capacitance value. The transformation of the impedance spectrum from a “low-resistance capacitive line” at low humidity to a “high-resistance *RC* semicircular arc” at high humidity signifies a change in the electrochemical relaxation time (*τ* = *RC*) of the system. Under high-frequency alternating electric fields, the excessive internal resistance barrier decelerates charge transport, preventing charges from penetrating into the microscopic interlayer regions within the composite film to participate in charging and discharging within the limited time frame. Numerous microscopic capacitors become “shielded” by the high-resistance barrier, transforming into electrically invisible dead zones, and the effective area *A* actually participating in energy storage contracts dramatically. According to the capacitance formula *C = εA/d*, the abrupt reduction in effective area *A* ultimately manifests as a sharp capacitance decay at the macroscopic level.

### 3.4. Applications

**Figure 6:**
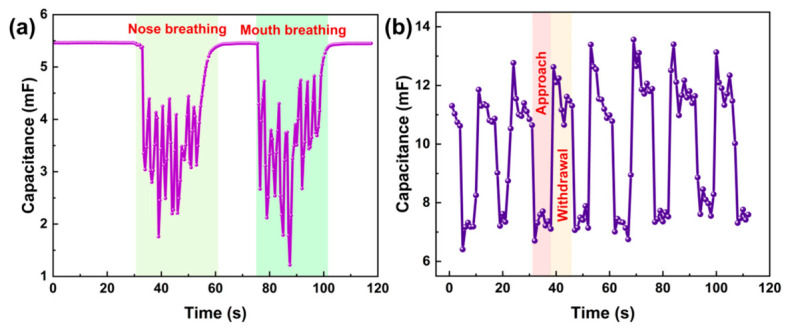
Demonstration of sensing capabilities for the GO-PEDOT:PSS-MXene@LIG humidity sensor. (**a**) Response behavior during nasal and oral exhalation monitoring. (**b**) Humidity response characteristics in non-contact sensing.

Based on the superior humidity-sensing performance of the GO-PEDOT:PSS-MXene@LIG humidity sensor, its potential applications in human respiration monitoring and non-contact detection are further investigated. Normal human breathing occurs through the nasal cavity, while certain conditions such as nasal congestion or high-intensity exercise induce a transition to oral respiration. Figure 6a demonstrates that the humidity sensor exhibits highly sensitive dynamic responses. Each individual inhalation and exhalation cycle generates distinct and regular humidity response peaks, with oral breathing producing significantly higher response amplitudes than nasal breathing. This phenomenon originates from the elevated RH of exhaled airflow caused by oral saliva. Furthermore, Figure 6b reveals that the sensor possesses a stable sensing capability for spatial non-contact interaction. When a finger repeatedly approaches and withdraws from the humidity sensor, the capacitive response displays regular alternating decreases and increases, with excellent curve reproducibility. These results fully confirm the reliability of this sensor for non-contact human–machine interaction applications.

## 4. Conclusions

This study successfully demonstrates a high-performance flexible capacitive humidity sensor by integrating a highly hydrophilic GO-PEDOT:PSS-MXene ternary composite with LIG interdigitated electrodes. Rather than exhibiting a traditional positive capacitive response, the sensor relies on a swelling-driven mechanism where moisture absorption causes significant volumetric expansion of the composite. This expansion substantially increases the interlayer spacing and physically decouples the internal 3D conductive network, driving a sharp decrease in the effective electrode area that dictates the sensor’s pronounced negative capacitive response. Consequently, the device achieves an exceptional sensitivity of 18,643.02 μF/%RH over a broad range of 11–97% RH, alongside a low hysteresis of 9.41% and rapid response/recovery times of 31.7 s and 11.2 s, respectively. These robust sensing characteristics enable the highly reliable differentiation of nasal and oral respiratory rhythms and facilitate responsive non-contact spatial interaction. By elucidating this microstructural decoupling mechanism and demonstrating easily scalable fabrication without vacuum deposition, this work provides a strong foundation for the future design of high-sensitivity flexible platforms for clinical diagnostics and wearable human–machine interfaces.

## Data Availability

The original contributions presented in this study are included in the article/Supplementary Material. Further inquiries can be directed to the corresponding author.

## Supplementary Materials

The following supporting information can be downloaded at: https://www.mdpi.com/article/10.3390/ma19122537/s1, Figure S1. Dimensional schematic and photograph of the predesigned interdigitated electrode; Figure S2. Saturated aqueous salt solutions prepared for the generation of different RH levels; Figure S3. Effect of varying MXene proportions on the RH sensing capabilities of GO-PEDOT:PSS-MXene@LIG humidity sensors; Figure S4. Hysteresis curve of the GO-PEDOT:PSS-MXene@LIG humidity sensor; Figure S5. Long-term stability, mechanical flexibility, and batch-to-batch reproducibility of GO-PEDOT:PSS-MXene@LIG humidity sensors. (a) Stability evaluation over extended operational periods. (b) Sensor response characteristics under different bending configurations at 58% RH. (c) Response consistency across three independent fabrication batches measured at 58% RH; Figure S6. Nyquist plots of the GO-PEDOT:PSS-MXene@LIG humidity sensor under varied RH levels; Table S1. Performance comparison of recently reported capacitive humidity sensors. References [40,41,42,43,44,45,46,47,48] are cited in Supplementary Materials.

## References

[1] Xiao C., Liu X., Zhao Y., Huang C., Zhou N., Mao H. (2025). Flexible humidity sensors for diverse applications. Microsyst. Nanoeng..

[2] Lahkar R., Dehingia B., Chouhan S., Kalita H. (2025). Flexible and Cost-Effective Graphene-Based Sensor on Paper Substrate Using Pencil IDEs for Multifunctional Applications in Plant and Human Health Monitoring. ACS Appl. Electron. Mater..

[3] Yang X., Lan L., Pan X., Di Q., Liu X., Li L., Naumov P., Zhang H. (2023). Bioinspired soft robots based on organic polymer-crystal hybrid materials with response to temperature and humidity. Nat. Commun..

[4] Zhang Z., Li J., Chen H., Wang H., Luo Y., Si R., Xie R., Tao K., Yang B.-R., Zhang D. (2025). Scalable Fabrication of Uniform Fast-Response Humidity Field Sensing Array for Respiration Recognition and Contactless Human-Machine Interaction. Adv. Funct. Mater..

[5] Li Z., Cheng Z., Wang Y., Zhang Z., Wu J. (2024). Single-layer graphene based resistive humidity sensor enhanced by graphene quantum dots. Nanotechnology.

[6] Li C., Xiong J., Zhao J. (2024). Oxygen plasma treatment-enhanced humidity sensing performance of MoS_2_ nanoparticles-anchored nitrogen-doped laser-induced graphene. Sens. Actuators B Chem..

[7] Jiang Y., Wu L., Chen Q., Li N., Tian J. (2025). High-performance capacitive humidity sensor based on flower-like SnS_2_/Ti_3_C_2_ MXene for respiration monitoring and non-contact sensing. Sens. Actuators B Chem..

[8] Li L., Zhang C., Xu Z., Gu L., Xu R., Zhao J. (2025). Synergistic MXene/GO composites for flexible capacitive humidity sensors with ultrahigh sensitivity and fast response. Surf. Interfaces.

[9] Liu J., Ng D.K.T., Koh Y., Samanta S., Xu L., Husni M.H.K.M., Srinivas M., Leotti A., Hur Y.J., Zhang Q. (2025). Piezoelectric micro diaphragm based high performance humidity sensor. Sens. Actuators B Chem..

[10] Guo J., Wen R., Liu Y., Zhang K., Kou J., Zhai J., Wang Z.L. (2018). Piezotronic Effect Enhanced Flexible Humidity Sensing of Monolayer MoS_2_. ACS Appl. Mater. Interfaces.

[11] Romero F.J., Rivadeneyra A., Salinas-Castillo A., Ohata A., Morales D.P., Becherer M., Rodriguez N. (2019). Design, fabrication and characterization of capacitive humidity sensors based on emerging flexible technologies. Sens. Actuators B Chem..

[12] Wang H., Liu X., Yu Y., Zhao J., Gao Y., Fei T., Chen Z. (2025). High-Performance Humidity Sensors Based on Hexagonal Boron Nitride Films Prepared by Magnetron Sputtering. IEEE Sens. J..

[13] Dong Y.F., Li L.Y., Jiang W.F., Wang H.Y., Li X.J. (2009). Capacitive humidity-sensing properties of electron-beam-evaporated nanophased WO_3_ film on silicon nanoporous pillar array. Phys. E.

[14] Lin J., Peng Z., Liu Y., Ruiz-Zepeda F., Ye R., Samuel E.L.G., Yacaman M.J., Yakobson B.I., Tour J.M. (2014). Laser-induced porous graphene films from commercial polymers. Nat. Commun..

[15] Le T.-S.D., Phan H.-P., Kwon S., Park S., Jung Y., Min J., Chun B.J., Yoon H., Ko S.H., Kim S.-W. (2022). Recent Advances in Laser-Induced Graphene: Mechanism, Fabrication, Properties, and Applications in Flexible Electronics. Adv. Funct. Mater..

[16] Aftab S., Koyyada G., Mukhtar M., Kabir F., Nazir G., Memon S.A., Aslam M., Assiri M.A., Kim J.H. (2024). Laser-Induced Graphene for Advanced Sensing: Comprehensive Review of Applications. ACS Sens..

[17] Huang L., Su J., Song Y., Ye R. (2020). Laser-Induced Graphene: En Route to Smart Sensing. Nano-Micro Lett..

[18] Pasalwad K.A., Baby N., Edjenguele A., Sadhasivam S., Palanisamy G., Magdum S.S., Thangarasu S., Oh T.H. (2025). Progress on polymer-based materials and composites for humidity sensor applications: From materials aspects to sensor performances. J. Mater. Chem. A.

[19] Guan X., Yu Y., Hou Z., Wu K., Zhao H., Liu S., Fei T., Zhang T. (2022). A flexible humidity sensor based on self-supported polymer film. Sens. Actuators B Chem..

[20] Mistry K., Nguyen V.H., Arabi M., Ibrahim K.H., Asgarimoghaddam H., Yavuz M., Muñoz-Rojas D., Abdel-Rahman E., Musselman K.P. (2022). Highly Sensitive Self-Actuated Zinc Oxide Resonant Microcantilever Humidity Sensor. Nano Lett..

[21] Kumar A., Gupta G., Bapna K., Shivagan D.D. (2023). Semiconductor-metal-oxide-based nano-composites for humidity sensing applications. Mater. Res. Bull..

[22] Waheed W., Anwer S., Khan M.U., Sajjad M., Alazzam A. (2024). 2D Ti_3_C_2_T_x_-MXene nanosheets and graphene oxide based highly sensitive humidity sensor for wearable and flexible electronics. Chem. Eng. J..

[23] Beniwal A., John D.A., Dahiya R. (2023). PEDOT:PSS-Based Disposable Humidity Sensor for Skin Moisture Monitoring. IEEE Sens. Lett..

[24] Ma H., Gao Q., Zhang Z., Yang K., Li J., Chen Y., Ding J., Zhang W., Fan X. (2026). Humidity sensing characteristics of graphene and MoS_2_ as well as their heterostructures with different stacking configurations. Nanoscale.

[25] Kuş M., Okur S. (2009). Electrical characterization of PEDOT:PSS beyond humidity saturation. Sens. Actuators B Chem..

[26] Zhang X., Zhang G., Wang F., Chi H. (2024). Evolution of Oxygen Content of Graphene Oxide for Humidity Sensing. Molecules.

[27] Song Y., Dan R., Li L., Xia X., Zhao J., Xu R. (2025). A flexible humidity sensor based on GO/PEDOT: PSS modified laser-induced graphene electrode. Sens. Actuators A Phys..

[28] Chaloeipote G., Wongchoosuk C. (2024). Flexible humidity sensor based on PEDOT:PSS/Mxene nanocomposite. Flex. Print. Electron..

[29] Ferrari A.C., Basko D.M. (2013). Raman spectroscopy as a versatile tool for studying the properties of graphene. Nat. Nanotechnol..

[30] Wahab H., Jain V., Tyrrell A.S., Seas M.A., Kotthoff L., Johnson P.A. (2020). Machine-learning-assisted fabrication: Bayesian optimization of laser-induced graphene patterning using in-situ Raman analysis. Carbon.

[31] Yan J., Ren C.E., Maleski K., Hatter C.B., Anasori B., Urbankowski P., Sarycheva A., Gogotsi Y. (2017). Flexible MXene/Graphene Films for Ultrafast Supercapacitors with Outstanding Volumetric Capacitance. Adv. Funct. Mater..

[32] Mikhraliieva A., Lima A.R.S., Jost C.L., Nazarkovsky M., Xing Y., Zaitsev V. (2024). Mesoporous Nitrogen-Doped Holey Reduced Graphene Oxide: Preparation, Purification, and Application for Metal-Free Electrochemical Sensing of Dopamine. Small.

[33] Eom W., Shin H., Han T.H. (2023). Tracking the thermal dynamics of Ti_3_C_2_T_x_ MXene with XPS and two-dimensional correlation spectroscopy. Appl. Phys. Lett..

[34] Zotti G., Zecchin S., Schiavon G., Louwet F., Groenendaal L., Crispin X., Osikowicz W., Salaneck W., Fahlman M. (2003). Electrochemical and XPS Studies toward the Role of Monomeric and Polymeric Sulfonate Counterions in the Synthesis, Composition, and Properties of Poly(3,4-ethylenedioxythiophene). Macromolecules.

[35] Luo Y., Zhu Q., Cao L., Fan L., Gu F., Xiong S. (2025). Aerosol Jet Printing of Hybrid Ti_3_C_2_T_x_ MXene/PEDOT:PSS Nanospheres for Flexible Planar/Fiber Architectured Micro-Supercapacitors. Adv. Eng. Mater..

[36] Miao J., Zhu Q., Li K., Zhang P., Zhao Q., Xu B. (2021). Self-propagating fabrication of 3D porous MXene-rGO film electrode for high-performance supercapacitors. J. Energy Chem..

[37] Wang Y., Zhang L., Zhou J., Lu A. (2020). Flexible and Transparent Cellulose-Based Ionic Film as a Humidity Sensor. ACS Appl. Mater. Interfaces.

[38] Borini S., White R., Wei D., Astley M., Haque S., Spigone E., Harris N., Kivioja J., Ryhänen T. (2013). Ultrafast Graphene Oxide Humidity Sensors. ACS Nano.

[39] Muckley E.S., Jacobs C.B., Vidal K., Mahalik J.P., Kumar R., Sumpter B.G., Ivanov I.N. (2017). New Insights on Electro-Optical Response of Poly(3,4-ethylenedioxythiophene):Poly(styrenesulfonate) Film to Humidity. ACS Appl. Mater. Interfaces.

[40] Lan L., Le X., Dong H., Xie J., Ying Y., Ping J. (2020). One-step and large-scale fabrication of flexible and wearable humidity sensor based on laser-induced graphene for real-time tracking of plant transpiration at bio-interface. Biosens. Bioelectron..

[41] Fei X., Huang J., Shi W. (2023). Humidity Sensor Composed of Laser-Induced Graphene Electrode and Graphene Oxide for Monitoring Respiration and Skin Moisture. Sensors.

[42] Li B., Tian Q., Su H., Wang X., Wang T., Zhang D. (2019). High sensitivity portable capacitive humidity sensor based on In_2_O_3_ nanocubes-decorated GO nanosheets and its wearable application in respiration detection. Sens. Actuators B.

[43] Yao X., Cui Y. (2020). A PEDOT:PSS functionalized capacitive sensor for humidity. Measurement.

[44] Romero F.J., Rivadeneyra A., Becherer M., Morales D.P., Rodríguez N. (2020). Fabrication and Characterization of Humidity Sensors Based on Graphene Oxide–PEDOT:PSS Composites on a Flexible Substrate. Micromachines.

[45] Li N., Jiang Y., Zhou C., Xiao Y., Meng B., Wang Z., Huang D., Xing C., Peng Z. (2019). High-Performance Humidity Sensor Based on Urchin-Like Composite of Ti_3_C_2_ MXene-Derived TiO_2_ Nanowires. ACS Appl. Mater. Interfaces.

[46] McGhee J.R., Sagu J.S., Southee D.J., Evans P.S.A., Wijayantha K.G.U. (2020). Printed, Fully Metal Oxide, Capacitive Humidity Sensors Using Conductive Indium Tin Oxide Inks. ACS Appl. Electron. Mater..

[47] Ganbold E., Sharma P.K., Kim E.-S., Lee D.-N., Kim N.-Y. (2023). Capacitive Humidity Sensor with a Rapid Response Time on a GO-Doped P(VDF-TrFE)/LiCl Composite for Noncontact Sensing Applications. Chemosensors.

[48] Strand E.J., Gopalakrishnan A., Crichton C.A., Palizzi M.J., Lee O., Borsa T., Bihar E., Goodrich P., Arias A.C., Shaheen S.E. (2025). Ultrathin Screen-Printed Plant Wearable Capacitive Sensors for Environmental Monitoring. Adv. Sens. Res..

