# Supplementary material for: A Flexible Capacitive Humidity Sensor Enabled by LIG-Anchored Synergistic GO-PEDOT:PSS-MXene Composite
Source: Materials (Basel). 2026 Jun 11;19(12):2537. doi: 10.3390/ma19122537 (PMC13303245; doi:10.3390/ma19122537)
Supplement: Supplementary file 1 [file materials-19-02537-s001.zip › materials-4349801-supplementary.pdf]

Supporting Information

A Flexible Capacitive Humidity Sensor Enabled by LIG-Anchored Synergistic GO-PEDOT:PSS-MXene Composite

*Jitong Ren, Ronghui Dan, Yanyan Guo, Jiang Zhao**

College of Integrated Circuit Science and Engineering, Nanjing University of Posts and Telecommunications, 9 Wenyuan Road, Nanjing 210023, P. R. China

*Corresponding author, E-mail: jzhao@njupt.edu.cn (J. Zhao).

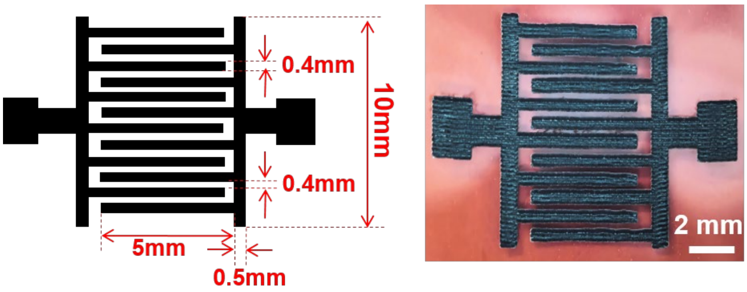

Figure S1. Dimensional schematic and photograph of the predesigned interdigitated electrode.

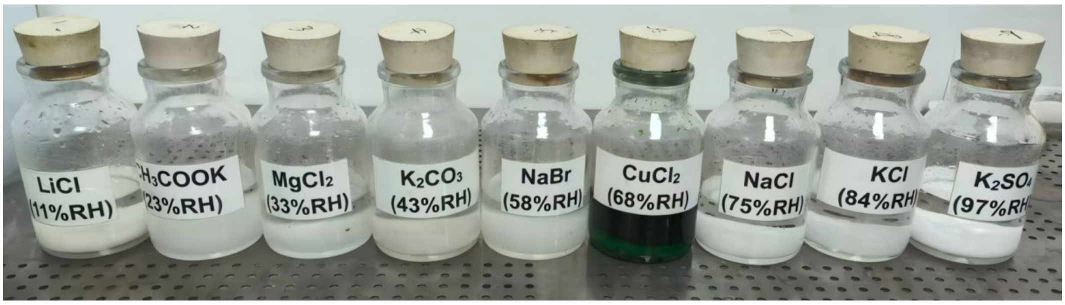

Figure S2. Saturated aqueous salt solutions prepared for the generation of different RH levels.

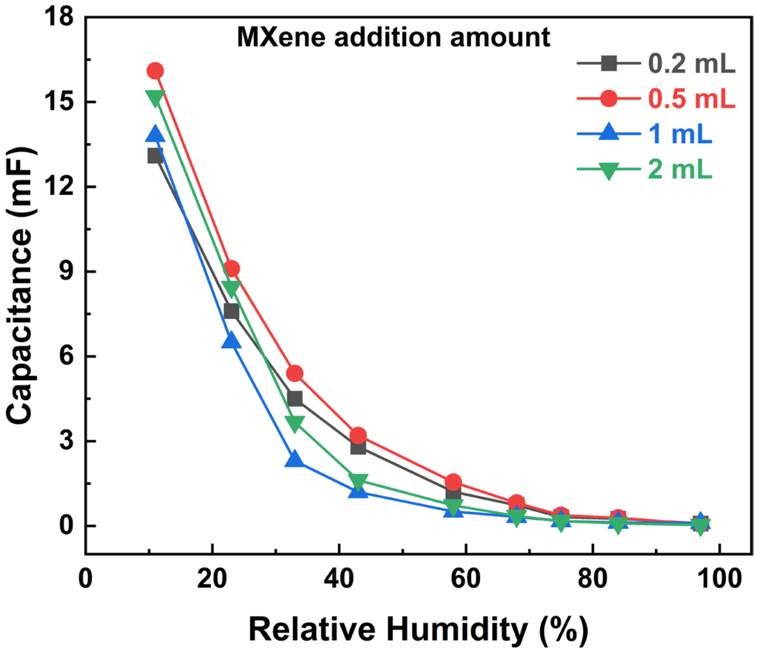

Figure S3. Effect of varying MXene proportions on the RH sensing capabilities of GO-PEDOT:PSS-MXene@LIG humidity sensors.

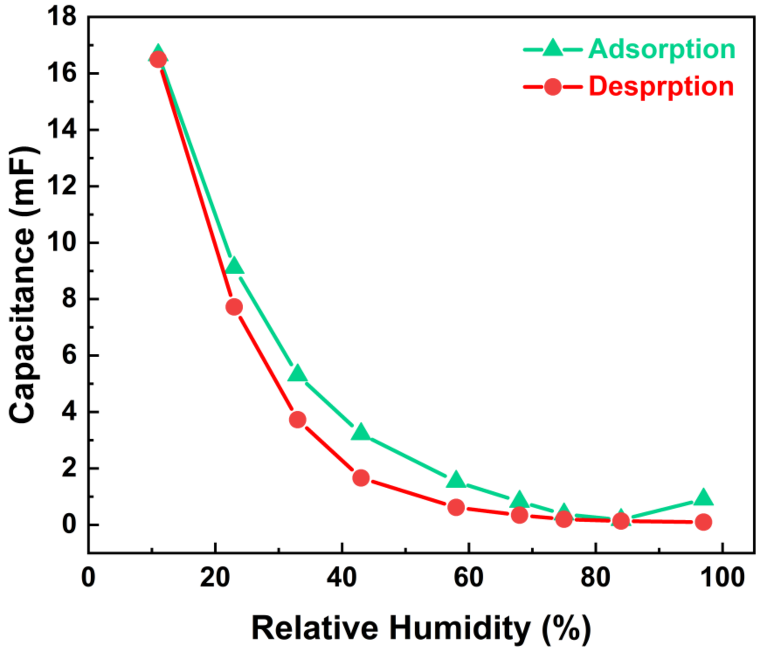

Figure S4. Hysteresis curve of the GO-PEDOT:PSS-MXene@LIG humidity sensor.

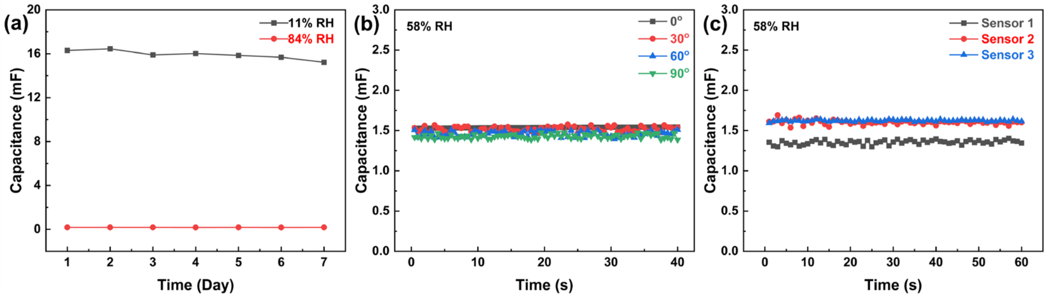

Figure S5. Long-term stability, mechanical flexibility, and batch-to-batch reproducibility of GO-PEDOT:PSS-MXene@LIG humidity sensors. (a) Stability evaluation over extended operational periods. (b) Sensor response characteristics under different bending configurations at 58% RH. (c) Response consistency across three independent fabrication batches measured at 58% RH.

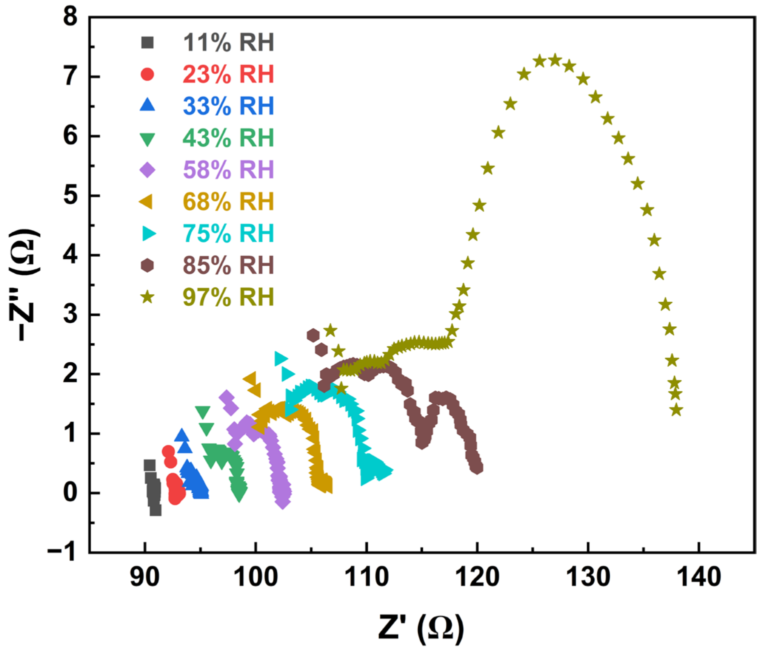

Figure S6. Nyquist plots of the GO-PEDOT:PSS-MXene@LIG humidity sensor under varied RH levels.

Table S1. Performance comparison of recently reported capacitive humidity sensors.

Humidity-sensing materials	Sensitivity (/%RH)	Detection range (%RH)	Response/recovery Time (s)	Ref.
GO	3215.25 pF	10–90	15.8	[1]
LIG/GO	3862 pF	10–90	58/15	[2]
GO/In_2O_3	1061.6 pF	11–97	15/2.5	[3]
PEDOT: PSS	0.05pF	52.0–93.4	<30/<60	[4]
GO/PEDOT:PSS	11.53 nF	20–90	701/496	[5]
Ti_3C_2/TiO_2	1614 pF	7–97	9.4/4.5	[6]
ITO/Al_2O_3	7.76 pF	5–95	47.2/49.5	[7]
GO-doped P (VDF-TrFE)/LiCl	1708.8 pF	25–93	7.8/4.8	[8]
Ethyl cellulose/carbon microparticle	1000 pF	1–85	–	[9]
GO-PEDOT: PSS-MXene	18643.02 μF	11–97	31.7/11.2	This work

References

1. Lan, L.; Le, X.; Dong, H.; Xie, J.; Ying, Y.; Ping, J., One-step and large-scale fabrication of flexible and wearable humidity sensor based on laser-induced graphene for real-time tracking of plant transpiration at bio-interface. *Biosens. Bioelectron.* **2020,** 165, 112360.
2. Fei, X.; Huang, J.; Shi, W., Humidity Sensor Composed of Laser-Induced Graphene Electrode and Graphene Oxide for Monitoring Respiration and Skin Moisture. *Sensors* **2023,** 23, (15), 6784.
3. Li, B.; Tian, Q.; Su, H.; Wang, X.; Wang, T.; Zhang, D., High sensitivity portable capacitive humidity sensor based on In_2O_3 nanocubes-decorated GO nanosheets and its wearable application in respiration detection. *Sens. Actuators, B* **2019,** 299, 126973.
4. Yao, X.; Cui, Y., A PEDOT:PSS functionalized capacitive sensor for humidity. *Measurement* **2020,** 160, 107782.
5. Romero, F. J.; Rivadeneyra, A.; Becherer, M.; Morales, D. P.; Rodríguez, N., Fabrication and Characterization of Humidity Sensors Based on Graphene Oxide–PEDOT:PSS Composites on a Flexible Substrate. *Micromachines* **2020,** 11, (2), 148.
6. Li, N.; Jiang, Y.; Zhou, C.; Xiao, Y.; Meng, B.; Wang, Z.; Huang, D.; Xing, C.; Peng, Z., High-Performance Humidity Sensor Based on Urchin-Like Composite of Ti_3C_2 MXene-Derived TiO_2 Nanowires. *ACS Appl. Mater. Interfaces* **2019,** 11, (41), 38116–38125.
7. McGhee, J. R.; Sagu, J. S.; Southee, D. J.; Evans, P. S. A.; Wijayantha, K. G. U., Printed, Fully Metal Oxide, Capacitive Humidity Sensors Using Conductive Indium Tin Oxide Inks. *ACS Appl. Electron. Mater.* **2020,** 2, (11), 3593–3600.
8. Ganbold, E.; Sharma, P. K.; Kim, E.-S.; Lee, D.-N.; Kim, N.-Y., Capacitive Humidity Sensor with a Rapid Response Time on a GO-Doped P(VDF-TrFE)/LiCl Composite for Noncontact Sensing Applications. *Chemosensors* **2023,** 11, (2), 122.
9. Strand, E. J.; Gopalakrishnan, A.; Crichton, C. A.; Palizzi, M. J.; Lee, O.; Borsa, T.; Bihar, E.; Goodrich, P.; Arias, A. C.; Shaheen, S. E.; McLeod, R. R.; Whiting, G. L., Ultrathin Screen-Printed Plant Wearable Capacitive Sensors for Environmental Monitoring. *Adv. Sens. Res.* **2025,** 4, (3), 2400177.
